# Expansion of human bone marrow-derived mesenchymal stromal cells with enhanced immunomodulatory properties

**DOI:** 10.1186/s13287-023-03481-7

**Published:** 2023-09-19

**Authors:** Shu Hui Neo, Zhisheng Her, Rashidah Othman, Ching Ann Tee, Li Ching Ong, Yuehua Wang, Irwin Tan, Jaylen Tan, Yanmeng Yang, Zheng Yang, Qingfeng Chen, Laurie A. Boyer

**Affiliations:** 1 Critical Analytics for Manufacturing of Personalized Medicine (CAMP), Interdisciplinary Research Group, Singapore-MIT Alliance for Research and Technology (SMART), 1 Create Way, Enterprise Wing, #04-13/14, Singapore, 138602 Republic of Singapore; 2 Institute of Molecular and Cell Biology (IMCB), Agency for Science, Technology and Research (A*STAR), 61 Biopolis Drive, Proteos, Singapore, 138673 Republic of Singapore; 3 Invivocue Pte Ltd, 51 Science Park Road, #01-11/13 The Aries, Singapore Science Park II, Singapore, 117586 Republic of Singapore; 4 Department of Orthopaedic Surgery, National University of Singapore, NUHS, 1E Kent Ridge RoadTower Block 11, Singapore, 119288 Republic of Singapore; 5 NUS Tissue Engineering Program, Life Sciences Institute, National University of Singapore, 27 Medical Drive, DSO (Kent Ridge) Building, Level 4, Singapore, 117510 Republic of Singapore; 6 Department of Microbiology and Immunology, Yong Loo Lin School of Medicine, National University of Singapore, 5 Science Drive 2, Singapore, 117545 Republic of Singapore; 7 Department of Biological Engineering, Massachusetts Institute of Technology, 77 Massachusetts Avenue, Cambridge, MA 02139 USA; 8 Department of Biology, Massachusetts Institute of Technology, 77 Massachusetts Avenue, Cambridge, MA 02139 USA

**Keywords:** Mesenchymal stromal cells, Immunomodulation, T cells, Cell culture, Confluency, Graft-versus-host disease

## Abstract

**Background:**

Mesenchymal stromal cells (MSCs) have broad potential as a cell therapy including for the treatment of drug-resistant inflammatory conditions with abnormal T cell proliferation such as graft-versus-host disease (GVHD). Clinical success, however, has been complicated by the heterogeneity of culture-expanded MSCs as well as donor variability. Here, we devise culture conditions that promote expansion of MSCs with enhanced immunomodulatory functions both in vitro and in animal models of GVHD.

**Methods:**

Human bone marrow-derived MSCs were expanded at high-confluency (MSC_HC_) and low-confluency state (MSC_LC_). Their immunomodulatory properties were evaluated with in vitro co-culture assays based on suppression of activated T cell proliferation and secretion of pro-inflammatory cytokines from activated T cells. Metabolic state of these cells was determined, while RNA sequencing was performed to explore transcriptome of these MSCs. Ex vivo expanded MSC_HC_ or MSC_LC_ was injected into human peripheral blood mononuclear cells (PBMC)-induced GVHD mouse model to determine their in vivo therapeutic efficacy based on clinical grade scoring, human CD45^+^ blood count and histopathological examination.

**Results:**

As compared to MSC_LC_, MSC_HC_ significantly reduced both the proliferation of anti-CD3/CD28-activated T cells and secretion of pro-inflammatory cytokines upon MSC_HC_ co-culture across several donors even in the absence of cytokine priming. Mechanistically, metabolic analysis of MSC_HC_ prior to co-culture with activated T cells showed increased glycolytic metabolism and lactate secretion compared to MSC_LC_, consistent with their ability to inhibit T cell proliferation. Transcriptome analysis further revealed differential expression of immunomodulatory genes including *TRIM29*, *BPIFB4*, *MMP3* and *SPP1* in MSC_HC_ as well as enriched pathways including cytokine–cytokine receptor interactions, cell adhesion and PI3K-AKT signalling_._ Lastly, we demonstrate in a human PBMC-induced GVHD mouse model that delivery of MSC_HC_ showed greater suppression of inflammation and improved outcomes compared to MSC_LC_ and saline controls.

**Conclusion:**

Our study provides evidence that ex vivo expansion of MSCs at high confluency alters the metabolic and transcriptomic states of these cells. Importantly, this approach maximizes the production of MSCs with enhanced immunomodulatory functions without priming, thus providing a non-invasive and generalizable strategy for improving the use of MSCs for the treatment of inflammatory diseases.

**Supplementary Information:**

The online version contains supplementary material available at 10.1186/s13287-023-03481-7.

## Background

Mesenchymal stromal cells (MSCs) have been widely investigated as a cell therapy product due to their low immunogenicity, robust ability to engraft and differentiation potential into osteoblasts, adipocytes and chondrocytes [1]. MSCs can be isolated from bone marrow [2], adipose tissue [3], placenta [4] and umbilical cord [5] making them a readily available cell type for clinical indications; however, their utility has been limited by donor variability and their unpredictable phenotypic heterogeneity during ex vivo expansion. For example, the yield of MSCs isolated from primary tissue is often low and requires expansion to reach sufficient numbers for therapy. A lack of standardized cell culture conditions including cell seeding density could introduce further heterogeneity in culture-expanded MSCs leading to differences in morphology, gene expression patterns and differentiation potency [6–8]. MSC enrichment is rarely performed after culture expansion before administration to a patient, which can also impact their therapeutic potency.

Growing evidence suggests that MSCs are a promising therapy for treating inflammatory conditions and diseases due to their immunomodulatory effects [9–14]. Studies show that MSCs can suppress inflammation upon migrating to sites of damage when infused in vivo [15]. Although the mechanisms of action are still unclear, MSCs can inhibit proliferation of activated T cells by promoting cell cycle arrest [9, 11, 16–18]. Consequently, immunomodulation of T cells by MSCs results in reduced secretion of the pro-inflammatory cytokines IFN-γ and IL-17A. Despite their potential, a lack of data connecting biological activity to therapeutic outcomes has limited the use of MSC for inflammatory diseases [19, 20] including graft-versus-host disease (GVHD) [21, 22] and rheumatoid arthritis [23, 24]. Thus, ex vivo expansion strategies that can enrich for MSCs with defined immunomodulatory functions have become a major focus to improve their use as cell therapy products.

Currently, priming MSCs with pro-inflammatory cytokines including TNF-α [25] and IFN-ɣ [26, 27] are used to improve their ability to suppress T cells and reduce inflammation. Cytokine priming strategies, however, have several disadvantages when considering downstream clinical applications. For example, priming can lead to increased immunogenicity of MSCs [28] and possible host rejection, reducing safety and efficacy. Priming can also introduce further heterogeneity during expansion given that different strategies employ various combinations and concentrations of cytokines [25, 29]. Moreover, the use of recombinant cytokines is costly and requires timely characterization due to batch differences and specific activities during production [30]. Finally, current methods that use unselected peripheral blood mononuclear cells (PBMCs) to study MSC-mediated immunomodulation could make it difficult to distinguish the direct effects of MSCs on T cells due to the presence of other immune cell types [18, 31–33].

Here, we show that culturing bone marrow-derived MSCs cultured at high confluency (MSC_HC_) without cytokine priming robustly enhanced the suppression of anti-CD3/CD28-activated T cell proliferation and the secretion of pro-inflammatory cytokines across multiple donors. MSC_HC_ conditions resulted in metabolic reprogramming towards glycolysis and to increased lactate production, properties known to limit T cell proliferation. Consistent with our observations, transcriptome analysis in MSC_HC_ revealed differential expression of genes and pathways associated with immunomodulation compared to MSC_LC_. Specifically, we identified enriched pathways related to immunomodulation such as cytokine–cytokine receptor interactions, cell adhesion and PI3K-AKT signalling in MSC_HC_ donors exhibiting the most robust immunosuppression in co-cultures. GVHD is a known challenge to allogenic hematopoietic stem cell transplant, and it primarily involves donor T cells in the graft attacking host tissues, such as skin, gastrointestinal tract and liver [34]. The standard care is often steroids, but about half of the patients develop steroid resistance [35]. Treatment of human PBMCs-induced GVHD mouse model with MSC_HC_ led to a marked reduction in GVHD symptoms as compared to MSC_LC_-treated mice. Specifically, we show that a single MSC_HC_ dose at the onset of symptoms resulted in a lower disease severity based on clinical grade scoring and reduced lymphocyte infiltration in major organs. Thus, our approach provides a generalizable and inexpensive strategy that can be readily adapted to improve the safety and efficacy of basal MSCs for the treatment of GVHD and potentially more broadly for inflammatory disorders.

## Methods

### BM-MSC culture

Human adult bone marrow-derived MSCs were obtained from healthy donors (n = 6) and commercially purchased from Lonza Pte. Ltd (Lonza PT-2501). Donor information of these 6 donors is given in Table 1.

**Table 1:** Description of MSC donors

Name	Source	Age	Sex
Donor 1	Human bone marrow	26 Y	M
Donor 2	Human bone marrow	24 Y	M
Donor 3	Human bone marrow	25 Y	M
Donor 4	Human bone marrow	25 Y	M
Donor 5	Human bone marrow	19 Y	F
Donor 6	Human bone marrow	33 Y	F

Bone marrow-derived MSCs from human donors. *Y* years, *M* male, *F* female

These cells were cultured in MSC expansion media (low-glucose Dulbecco’s modified Eagle medium (DMEM) (Gibco) supplemented with 10% foetal bovine serum (FBS) (Gibco) and 1% penicillin/streptomycin (Gibco). All cells were thawed from frozen vials and subjected to recovery. They were routinely maintained in a humidified 37˚C incubator with 5% CO_2_, at a seeding density of 200 cells/cm^2^ (low confluency) or 3000 cells/cm^2^ (high confluency). Culture media was changed every 3 days and were harvested at same time using 0.05% Trypsin/EDTA solution (Life Technologies). The cell count and viability were determined using disposable haemocytometer (INCYTO, KR) and Trypan Blue (Thermo Fisher Scientific, SG). Cell confluency was quantified from a set of phase contrast microscopy images taken at 4× magnification (Olympus SC30) using a ImageJ algorithm (SMART CAMP). Cells harvested at end of passage 4 were used for all downstream experiments in this study.

### Immunophenotyping of MSCs by flow cytometry

Upon trypsinization and washing in phosphate-buffered saline (PBS), phenotypic expression of cell surface markers was performed on BM-MSCs with flow cytometry using the following fluorochrome-conjugated anti-human antibodies: CD73-fluorescein isothiocyanate (FITC), CD90-phycoerythrin (PE), CD105-allophycocyanin (APC), CD34-Brilliant Violet (BV) 510 and CD45-peridinin chlorophyll protein (PerCP) Cy5.5 (BD Biosciences). Positively stained cell populations were identified by comparing with fluorescence-minus-one (FMO) controls. Acquisition was performed using a CytoFLEX flow cytometer (Beckman Coulter) and analysed using FlowJo V10.

### In vitro multi-lineage differentiation of MSCs

MSCs were seeded at density of 3 × 10^4^ cells/well and 6 × 10^4^ cells/well for osteogenesis and adipogenesis, respectively, in 24-well plates for overnight attachment. The MSC expansion media was removed the following day and replaced with specific differentiation media (STEMCELL Technologies). Differentiation media was changed every 3 days for 14 days before processing. Osteogenesis induction was confirmed with Alizarin Red S staining (ScienCell) for calcium deposits while adipogenic differentiation was determined with Oil Red O staining (Sigma) for detection of lipid droplets according to manufacturer’s protocol. The subsequent dye extraction of Oil Red O and Alizarin Red S was performed for measurement of optical densities (OD) at 405 nm and 510 nm, respectively, using a plate reader (Tecan). Quantification of adipogenesis and osteogenesis was calculated by the measured OD of stain divided by the seeded cell number of each sample, represented as the normalized OD.

For chondrogenesis, MSCs were pelleted at 0.5 × 10^6^ cells/pellet in a 15-ml tube by centrifugation at 300*g*, 5 min. Chondrogenic media (STEMCELL Technologies) was added to this pellet and media was changed every 3 days for 3 weeks before glycosaminoglycan quantification. Hydrogel constructs were fixed in 10% neutral buffered formalin (Sigma-Aldrich) overnight at 4 °C followed by graded ethanol dehydration (70%, 70%, 80%, 80%, 95%, 95% ethanol at 15 min each) and 3 min of incubation with eosin (Sigma-Aldrich) for easy visualization during tissue sectioning. The samples were then washed with 100% ethanol thrice and dehydrated further in 100% ethanol for 20 min twice and HistoChoice® clearing agent (Sigma-Aldrich) for 20 min twice. The dehydrated samples were incubated in paraffin bath for 1 h before embedding in paraffin. Samples were cut into 5 µm section using microtome (Leica, USA) and collected on silane-coated slides (Thermo Scientific, USA). Tissue sections were de-waxed and rehydrated in xylene and graded ethanol, respectively. Immunohistochemical staining was used to identify Type II Collagen using Ultra Vision detection kit (Thermo Fisher Scientific, SG). The rehydrated sections were first blocked with hydrogen peroxide block for 15 min before pepsin treatment for 20 min at 37 °C. After which, the sections were treated with Ultra V Block for 5 min and subsequently with 1-h primary antibody incubation using Type II Collagen mouse monoclonal antibodies (Clone 6B3 at 1:500 dilution, Sigma-Aldrich). It was followed by secondary antibody incubation with biotinylated goat anti-mouse secondary antibody for 30 min. Streptavidin peroxidase was then added and incubated for 45 min before 1.5 min of incubation with DAB chromogen. Lastly, the sections were counterstained with hematoxylin, dehydrated and mounted for imaging.

### In vitro T cell assay

MSCs were seeded at 5 × 10^4^ cells/well in 48-well plate overnight before assay. PBMCs from a single donor were commercially purchased (STEMCELL Technologies) with details in Table 2.

**Table 2:** Description of PBMC donor

Name	Source	Age	Sex
Donor 1	Human peripheral blood	26 Y	M

Peripheral blood mononuclear cells from human donor. *Y*: years, *M*: Male

Negative immunoselection was performed to isolate CD3^+^ T cells using the EasySep™ Human T Cell Isolation Kit (STEMCELL Technologies) according to the manufacturer’s instructions. The purity of CD3^+^ T cells was more than 95%. CD3^+^ T cells were stained with Celltrace Violet (CTV) dye (Thermo) and stimulated with ImmunoCult™ Human CD3/CD28 T cell activator (STEMCELL Technologies) before direct co-culture with MSCs for 5 days at 1:3 (MSC: T cell ratio). These activators contain anti-human CD3 monospecific antibody complex and anti-human CD28 monospecific antibody complex. The co-culture media was collected at the end of day 5 of co-culture and spun at 1000 g, 5 min to retrieve the cell pellet (T cells) while the supernatant was kept in − 80 °C until cytokine analysis. Flow cytometric analysis was performed on harvested T cells using fluorochrome-conjugated anti-human antibodies: CD3-APC, CD4-BV605, CD45-PerCP Cy5.5, CD8a-PE-Cy7 (BD Biosciences). Positively stained cell populations were identified by comparing with FMO controls. Acquisition was performed using a CytoFLEX flow cytometer (Beckman Coulter Life Sciences) and analysed using FlowJo V10. Quantification of T cell proliferation was determined using the formula [36] to determine proliferative index (PI): PI = Log[FI_nd_/MFI_all_]/Log[2], with MFI_all_ = median fluorescence intensity of all viable T cells and FI_nd_ = peak fluorescence intensity of the viable non-divided cells.

### Enzyme-linked immunosorbent assay (ELISA)

The concentration of cytokines, including IFN-ɣ and IL-17A, was determined by Luminex^®^-based multiplex assays (R&D Systems) according to manufacturer’s instructions. All samples were analysed in duplicate and repeated for 6 different donors. The data were obtained with a MAGPIX reader (Millipore), and concentrations were derived from measured mean fluorescence intensities using fitted standard curves using 5-parameter logistic regression (SSL5) using the Milliplex Analyst software (Millipore).

### Measurement of cell metabolism

MSCs were seeded at 2 × 10^4^ cells/well in a Seahorse XF24 V7-PS Microplate (Agilent Technologies) overnight. Cell culture media was collected the next day for measurement of lactate concentration using Cedex Bio Analyzer (Roche). Lactate production rate was calculated by deducting the lactate concentration in base media and normalized to total cell number and time of culture. MSCs (2.5 × 10^4^ cells/well) seeded on Seahorse 24-well microplate were used to measure extracellular acidification rate (ECAR) using Seahorse XF24 Extracellular Flux Analyzer (Agilent Technologies) according to manufacturer’s protocols.

### RNA isolation and sequencing

Total RNA was isolated using RNeasy Mini Kit (Qiagen). RNA libraries were prepared using TruSeq Stranded mRNA Library Prep Kit according to manufacturer’s protocol. Transcriptome sequencing was performed using Illumina Novaseq 6000 system. The raw data were analysed using Partek^®^ Flow^®^ software (Partek Inc., St. Louis, MO, USA). Reads were mapped to the Genome Reference Consortium Human Reference 38 (hg38) using the STAR aligner. The aligned reads were quantified and annotated based on Ensembl transcripts (release 108). ANOVA was performed as the statistical method for differential expression analysis, and differentially expressed genes (DEGs) were identified by filtering with two criteria for significance: magnitude of fold change (FC) ≥ 2 and false discovery rate step-up ≤ 0.05. Heatmaps were generated using Partek® Flow® software with genes mapped according to their respective *z*-score. Significantly enriched pathways were identified with the Kyoto Encyclopedia of Genes and Genomes Pathways (*Homo sapiens*) (KEGG) database, with the plot of significantly enriched pathways (*p* ≤ 0.05) generated by R Studio.

### In vivo testing with human PBMC-induced mouse GVHD model

Female immunodeficient mice NOD-SCID IL2rg^null^ (NSG) mice were purchased from the Jackson Laboratory (stock number 005557). All mice were bred and kept under pathogen-free conditions in Biological Resource Centre, Agency for Science, Technology and Research, Singapore (A*STAR) on controlled 12-h light–dark cycle with ad libitum access to normal diet and drinking water. All experiments and procedures were approved by the Institutional Animal Care and Use Committee of the Agency for Science, Technology and Research, Singapore, in accordance with the guidelines of the Agri-Food and Veterinary Authority and the National Advisory Committee for Laboratory Animal Research (NACLAR) of Singapore. Eight- to ten-week-old mice were sub-lethally irradiated at 1 Gy and engrafted with 5 × 10^6^ human PBMCs (STEMCELL Technologies) via tail vein intravenously, 24 h after irradiation. The mice were monitored daily for GVHD symptoms, and blood was withdrawn weekly on Day 17, 24, 28, 35, 42 and 49 post-PBMC injection to evaluate human CD45^+^ cells (hCD45) reconstitution using flow cytometry. Based on the reconstitution analysis on Day 17, mice with successful hCD45 engraftment (> 200 hCD45 counts per µl blood) were selected for subsequent evaluation of MSC therapeutic efficacy on GVHD. Mice were stratified based on their hCD45 counts and randomized using blocking method to either MSC_HC_, MSC_LC_ or saline groups. For the evaluation of MSC therapeutic efficacy on GVHD, each mouse was injected intraperitoneally with 3 x 10^6^ culture-expanded MSCs (MSC_HC_ or MSC_LC_) diluted in saline (0.9% sodium chloride; B.Braun) on Day 21 post-PBMC injection. Mice injected intraperitoneally with equal volume of saline were included as controls. Mice were injected intraperitoneally with culture-expanded MSCs, 21 days after PBMCs injection. The mice were monitored daily for GVHD symptoms daily, and blood was withdrawn weekly on Day 17, 24, 28, 35, 42 and 49 post-PBMC injection to evaluate hCD45 reconstitution using flow cytometry. The extent of GVHD severity was scored 0–2 based on (a) fur texture: 0 (normal), 1 (mild ruffling) and 2 (severe ruffling); (b) activity/movement: 0 (normal), 1 (mild decrease) and 2 (severe decrease); (c) posture: 0 (normal), 1 (hunch at rest) and 2 (severe hunching); (d) body weight loss: 0 (< 10%), 1 (10–25%) and 2 (> 25%); and (e) skin integrity: 0 (normal), 1 (scaling of paw/tail) and 2 (obvious denuded).

### Immunophenotyping of peripheral blood by flow cytometry

Peripheral blood was collected submandibularly from mice in K3EDTA minicollect^®^ tubes (Grenier Bio-One) at intermediate time points and endpoint. Red blood cells (RBCs) were lysed using RBC lysis buffer (Life Technologies). Live immune cells from peripheral blood were determined by staining with live/dead fixable blue dead cell stain kit (Life Technologies) for 10 min at room temperature prior to staining with mouse and human Fc receptor blocking reagents (Biolegend) for 10 min at room temperature to prevent non-specific binding. Subsequently, cells were stained with fluorescent-labelled surface markers which include anti-human CD45 (HI30; BD Biosciences) and anti-mouse CD45.1 (A20; Biolegend) for 30 min at 4 °C in the dark. After staining, cells were washed and resuspended in FACS buffer containing PBS, 0.2% bovine serum albumin (GE Healthcare Life Sciences) and 0.05% sodium azide (Merck) for flow cytometry data acquisition. Data were acquired using a LSR Fortessa X20 flow cytometer (BD Biosciences) with FACSDiva software and analysed using FlowJo software (version 10; Tree Star Inc). The absolute count of each immune cell type is determined using CountBright Counting Beads (Thermo Fisher Scientific) following manufacturer’s instructions.

### Hematoxylin and eosin (H&E) staining

Mice were euthanized using carbon dioxide (CO_2_) inhalation at endpoint. Lungs, colon, liver, kidneys and skin were removed from euthanized mice and fixed in 10% formalin. Fixed tissues were embedded in paraffin wax, processed to obtain 5 μm sections and subjected to Hematoxylin & Eosin (H&E) (Sigma) staining following established protocols. Histopathological images were acquired using Axio Scan. Z1 slide scanner (Zeiss) and analysed using Zen 2 (blue edition; Zeiss) software.

### Data processing

Data are presented as the means ± standard error of the mean (SEM) and are representative of at least three independent experiments. Statistical analyses for comparison between selected pairs were performed with Mann–Whitney test, Wilcoxon matched-pairs signed-rank test or one-way ANOVA (Tukey's multiple comparison test) as indicated using Prism v8 (GraphPad Software).

## Results

### Cell culture confluency does not impact MSC multi-lineage differentiation

Previous studies showed that cell culture confluency can affect MSC attributes; however, few donors have been systematically tested in any study limiting data interpretation [8, 37, 38]. To test the effect of cell confluency on MSC immunomodulatory functions, bone marrow-derived MSCs were expanded from 6 donors (Table 1) using the same culture conditions while varying only seeding density.

**Fig. 1:**
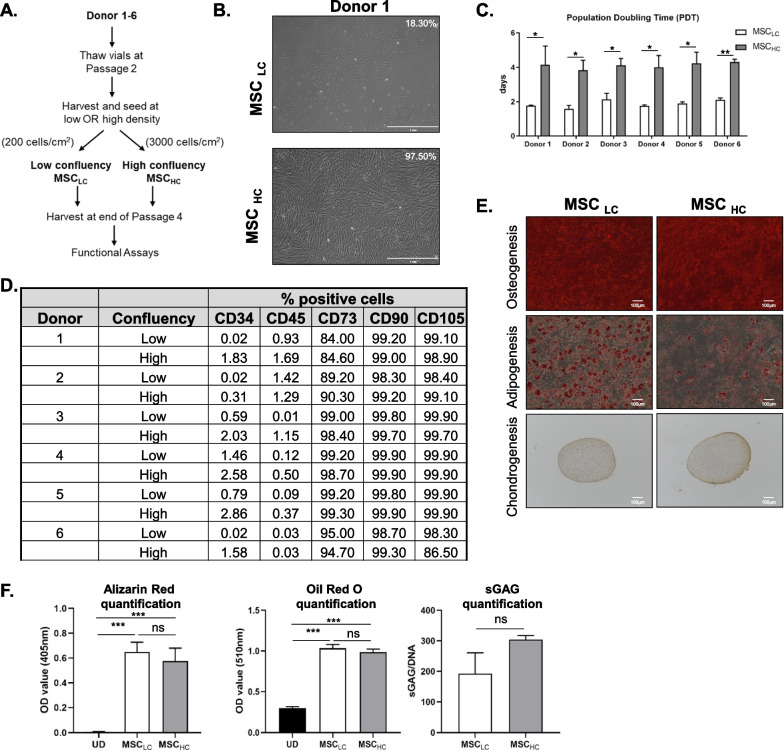
Phenotypic characterization of bone marrow-derived MSCs in this study. **A** Method for varying low (MSC_LC_) and high confluency (MSC_HC_) of BM-MSCs from 6 donors. **B** Representative images of MSC_LC_ and MSC_HC_ for Donor 1 (scale bar: 1 mm). **C** Population doubling time of MSC_LC_ and MSC_HC_ for Donor 1 to 6. **D** Flow cytometry-mediated characterization of surface markers to determine % positive BM-MSCs in each experimental condition for this study. **E** In vitro differentiation of MSC_LC_ and MSC_HC_ from Donor 1. Alizarin red staining for calcium deposits detected in osteoblasts, Oil red O staining for oil droplets in adipocytes and collagen II staining was performed for chondrocytes. Scale bar: 100 μm. **F** Differentiation assays performed in technical triplicates and quantified for Donor 1 as compared to undifferentiated cells (UD). One-way ANOVA (Tukey's multiple comparison test) or Mann–Whitney test was performed between selected pairs as statistical test. *p ≤ 0.05; **p ≤ 0.01 *** *p* ≤ 0.001; ns represents non-significant

MSCs were then expanded for 7 days in the absence of priming cytokines prior to harvesting for co-culture or downstream analysis (Fig. 1A). Specifically, BM-MSCs were seeded at 200 cells/cm^2^ and 3000 cells/cm^2^ resulting in ~ 19% confluency (MSC_LC_) or ~ 90% confluency (MSC_HC_) at day 7, respectively, as quantified with an imaging-based algorithm (Fig. 1B and Additional file 1: Fig. S1). MSC_HC_ proliferated slower and had a longer population doubling time (~ 4 days) compared to MSC_LC_ (~ 2 days) in all 6 donors (Fig. 1C), consistent with contact dependent cell cycle withdrawal [39].

To determine the effect of seeding density on MSC quality, we next analysed immunophenotypic cell surface antigens endorsed by the International Society of Cell and Gene Therapy (ISCT) [40]. Both MSC_LC_ and MSC_HC_ exhibited positive staining for CD73, CD90 and CD105, and minimal staining for hematopoietic markers CD34 and CD45 after 7 days in culture (Fig. 1D, Additional file 2: Fig. S2). The expression of CD34 and CD45 on bone marrow-derived MSCs has been controversial (41), which could explain their variability of these markers in our data.

**Fig. 2:**
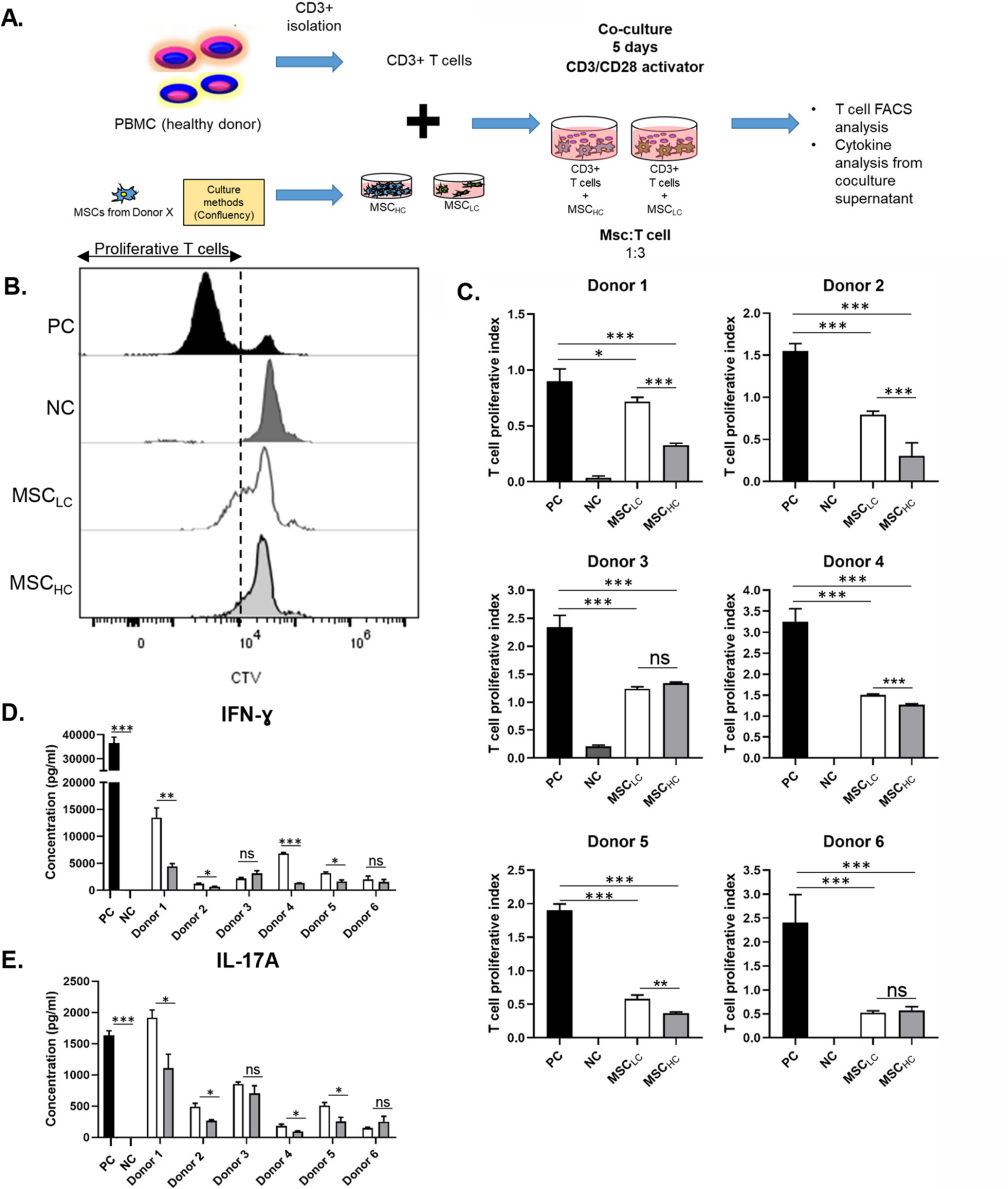
Inhibition of T cells proliferation in the presence of co-culture with MSC_LC_ or MSC_HC_. **A** CD3^+^ T cells were isolated from human PBMCs and stained with Celltrace Violet (CTV) proliferation dye to track T cell proliferation. Co-culture of T cells with MSC_LC_ or MSC_HC_ from Donor 1 to 6 was performed with CTV-stained T cells and anti-CD3/CD28 antibodies (Abs). **B** Representative histogram plots of CD3^+^ T cell proliferation in co-culture with MSCs, assessed by CTV dilution. **C** The extent of T cell proliferation was determined with T cell proliferative index (PI) calculated from their respective histogram by the following formula: PI = Log[FI_nd_/MFI_all_]/Log[2], with MFI_all_ = median fluorescence intensity of all viable T cells and FI_nd_ = peak fluorescence intensity of the viable non-divided cells. White bar indicates co-culture of MSCs harvested at low confluency (MSC_LC_) with T cells; light grey bar indicates co-culture of MSCs harvested at high confluency (MSC_HC_) with T cells; black bar indicates positive control (PC) containing anti-CD3/CD28-activated human CD3^+^ T cells without MSCs; dark grey bar indicates negative control (NC) containing naïve CD3^+^ T cells. The concentration of **D** IFN-ɣ and **E** IL-17A secreted into the co-culture media were measured by ELISA. Data shown were expressed as mean ± SEM of triplicates of separate experiments, representative of 6 donors. One-way ANOVA (Sidak’s multiple comparison test) was performed between selected pairs in each donor. *p ≤ 0.05; ** *p* ≤ 0.01 *** *p* ≤ 0.001; ns represents non-significant

To assess MSC differentiation potential in both conditions, we induced tri-lineage differentiation. Osteogenesis and adipogenesis were induced for 2 weeks as described [42, 43] and cultures were stained for Alizarin Red S and Oil Red O staining, respectively. We observed positive staining of calcium-containing differentiated osteocytes and lipid droplet-containing differentiated adipocytes from all donor MSCs independent of cell seeding density. To assess chondrogenesis, the same number of MSCs from both conditions were subjected to a 3D culture pellet assay in the presence of chondrogenic differentiation media for 3 weeks [44]. We observed both soluble glycosaminoglycans (sGAGs) and type II collagen in the pericellular matrix of differentiated MSCs across all donors in both conditions (Fig. 1E, F, Additional file 3: Fig. S3A, B). Together, these data indicate that culture confluency does not affect the quality or multi-lineage differentiation potential of donor MSCs.

**Fig. 3:**
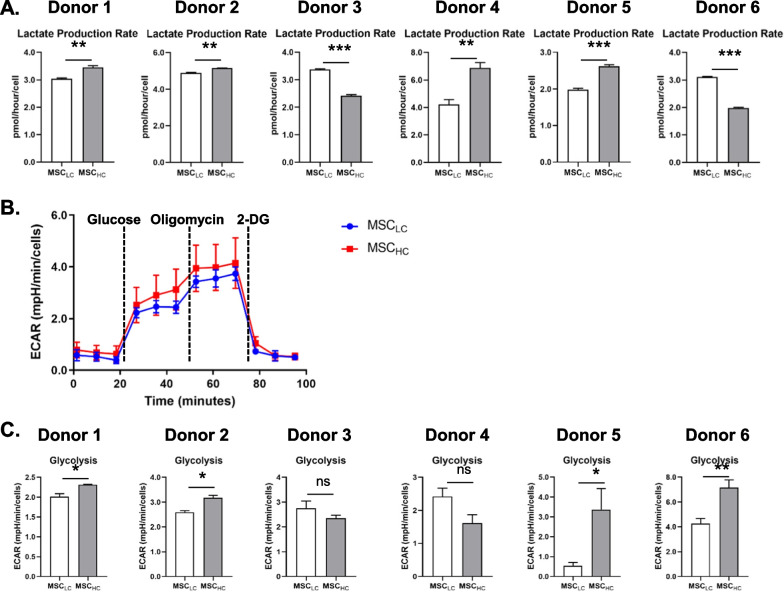
MSC_HC_ produced more lactate than MSC_LC_. **A** Lactate production rate were obtained from Cedex measurements and displayed for 6 donors. **B** Cell number-normalized ECAR profile plots of MSC_LC_ and MSC_HC_ from Donor 1 in response to glucose, oligomycin and 2-deoxyglucose (2-DG) in glycolysis stress test. **C** Basal glycolysis was calculated as (maximum rate measurement before Oligomycin injection)-(last rate before Glucose injection) for each donor. Data shown were expressed as mean ± S.E.M. of three measurements, from two independent experiments. Unpaired t-test (two-tailed) was performed. *p ≤ 0.05; **p ≤ 0.01; ns represents non-significant

### MSC_HC_ promotes enhanced suppression of anti-CD3/CD28-activated T cells in co-culture

MSCs seeded at high density relative to the number of PBMCs in co-culture assays can modestly inhibit phytohaemagglutin A (PHA)-stimulated PBMC proliferation [31]. These data suggest that an increased ratio of MSCs to stimulated PBMCs could impact immunomodulation during ex vivo expansion, but the target cell affected by MSC co-culture was not characterized in this study. To specifically test whether MSC confluency affects T cell proliferation, we harvested MSCs after 7 days in culture and used equal numbers of MSC_LC_ and MSC_HC_ for co-culture with anti-CD3/CD28-activated T cells (Fig. 2A)_._ We first isolated CD3^+^ T cells from PBMCs using negative immunoselection achieving > 90% purity (Additional file 4: Fig. S4A and Table S2).

**Fig. 4:**
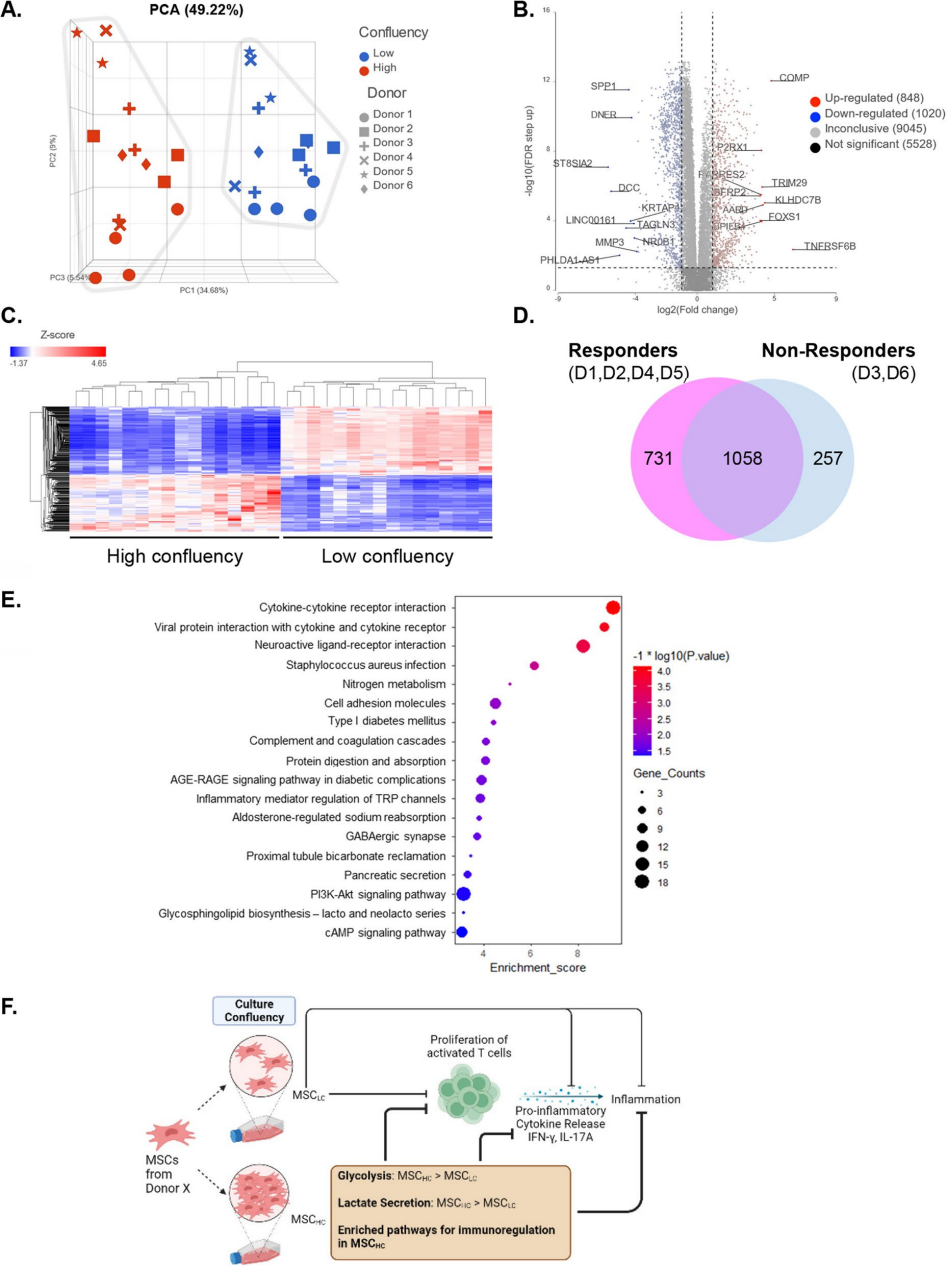
Differential gene expression analysis of MSC_HC_ and MSC_LC_. **A** Principle component analysis (PCA) plot show 2 clusters among datasets of MSC_HC_ and MSC_LC_ in 6 donors, with at least 2 technical replicates for each donor. **B** Volcano plot with top 10 up-regulated and down-regulated differentially expressed genes (DEGs) by comparing MSC_HC_ over MSC_LC_ for all donors measured by RNA-seq analysis. **C** Unsupervised hierarchical clustering using average linkage with Euclidean distance for DEGs. **D** Venn diagram showing number of significant DEGs found in respective section. **E** Significantly enriched signalling pathways (ranked by p-value) associated with 731 DEGs unique in responders (D1, D2, D4, D5). **F** Proposed model for establishing the relationship between MSC culture confluency and MSC-mediated immunosuppression on T cells. Created with BioRender.com

Flow cytometry showed that enriched cells comprise 58.8% and 36.6% of CD4^+^ T cells and CD8^+^ T cells, respectively (Additional file 4: Fig. S4B). We then activated the T cells with monoclonal antibodies against CD3/CD28 to mimic physiological stimulation of naïve T cells [18, 33, 45], in contrast to polyclonal stimulation by the plant lectin PHA [11]. Activated T cells were co-cultured at the same ratio with either MSC_LC_ or MSC_HC_ (1:3 MSC/T cell) for 5 days across all six donors. We examined T cell proliferation after 5 days of co-culture based on established guidelines for short term lymphocyte assays [31, 46–48].

We first quantified the T cell proliferation index (PI) at day 5 after co-culture based on the median fluorescent intensity (MFI) from the histogram representing the CTV-stained T cells for each sample (Fig. 2B) (36). Our data show that co-culture of anti-CD3/CD28-activated T cells with MSCs led to significant suppression of proliferation in all donors as compared to activated T cells alone (positive control/PC), whereas naïve T cells showed no proliferation (negative control/NC) (Fig. 2C). Notably, co-culture with MSC_HC_ resulted in significantly greater T cell suppression, with an average decrease of 1.90 ± 0.3165 in T cell PI compared to MSC_LC_ in 4 of the 6 donors. Notably, our analysis shows that MSCs cultured at high confluency retain enhanced immunomodulatory capacity for at least 5 days after harvesting even in the absence of cytokine priming.

### MSC_HC_: T cell co-cultures secrete reduced pro-inflammatory cytokines

Given the enhanced suppression of MSC_HC_ on T cell proliferation, we next measured the levels of pro-inflammatory cytokines (IFN-ɣ and IL-17A) typically secreted by activated T cells in vivo [49, 50]. Media was collected from all co-culture conditions and donors at day 5 and cytokines were quantified using a bead-based Luminex assay in a replicate set of experiments. We found that all pro-inflammatory cytokines tested showed significant reduction upon co-culture with MSCs compared to activated T cells alone (positive control/PC) (Fig. 2D, E). Notably, co-culture of MSC_HC_ and T cells led to a significant decrease in cytokine levels compared to MSC_LC_ for the same 4 donors that showed stronger inhibition of T cell proliferation. Our data suggest secretion of these pro-inflammatory cytokines correlates with T cell proliferation and that their levels can be more robustly modulated by MSCs cultured at high confluency.

### MSC_HC_ displays increased lactate secretion and glycolytic flux

Lactate is produced by cells that are highly glycolytic and can affect proliferation by inhibiting oxidative phosphorylation [51–53]. Indeed, increased lactate in the media can suppress T cell proliferation and cytokine production [52, 54–56]. Improved immunomodulatory capacity also correlates with shift towards glycolytic metabolism and lactate secretion upon priming naïve MSCs with IFN-ɣ or exposure to hypoxic conditions [56, 57]. To determine how MSC confluency affects lactate secretion from MSCs, we measured the lactate concentrations in the media in all conditions and donors before co-culture with T cells using a Cedex Bio Analyzer. Notably, MSCs derived from donors 1, 2, 4 and 5 that showed the highest T cell suppression activity in co-culture also showed increased lactate secretion (Fig. 3A). Using the Seahorse Flux Analyzer, we monitored the extracellular acidification rate (ECAR) measurements as an indicator of glycolysis (Fig. 3B) and showed that MSC_HC_ displayed higher basal glycolysis compared to MSC_LC_ in 4 of 6 donors (Fig. 3C). Although we observed a similar trend, the changes in ECAR did not correlate with changes in lactate secretion levels in some donors. It is important to note that ECAR measures the flux of protons, which can be contributed by other sources of extracellular acidification and not solely due to lactate secretion [58]. Taken together, our data demonstrate that expansion of MSCs at high density prior to co-culture results in altered metabolic states that correlate with enhanced immunomodulation across multiple donors.

### MSC_HC_ displays distinct transcriptome profile from MSC_LC_

To further investigate possible mechanisms, we performed RNA-seq analysis on MSC_LC_ and MSC_HC_ across all donors in a triplicate set of experiments. Principle component analysis (PCA) showed high concordance between low- and high-confluency states across the 6 donors (Fig. 4A). Among the 16,441 genes analysed, 1868 differentially expressed genes (DEGs) were detected between MSC_HC_ and MSC_LC_, with 848 DEGs up-regulated and 1020 DEGs down-regulated in MSC_HC_, based on a magnitude fold change of > 2.0 and false discovery rate ≤ 0.05 (Additional file 7: Table S1). The top 10 up-regulated DEGs (*TNFRSF6B, COMP, KLHDC7B, AARD, TRIM29, BPIFB4, P2RX1, RARRES2, FOXS1 and SFRP2*) and top 10 down-regulated genes (*ST8SIA2, DCC, PHLDA1-AS1, TAGLN3, SPP1, KRTAP4-9, DNER, LINC00161, NR0B1 and MMP3*) are labelled on the volcano plot (Fig. 4B). Heatmap analysis of the 1868 DEGs revealed distinct transcriptomic profiles for MSC_HC_ and MSC_LC_ in these six donors (Fig. 4C).

Because we wanted to identify potential pathways that characterize immunomodulation potency, we next categorized the donors into two groups based on MSC_HC_ suppression of T cell proliferation (1) responders: Donor 1, Donor 2, Donor 4, Donor 5; and (2) non-responders: Donor 3 and Donor 6. By performing DEG analysis of high confluency versus low confluency in both groups, we identified 731 DEGs that are unique to the responders (Additional file 8: Table S2), 257 DEGs that are unique to the non-responders and 1058 DEGs that are shared between the two groups (Fig. 4D). From the 731 DEGs, we performed pathway enrichment analysis and identified several biological pathways (p ≤ 0.05) associated with immunomodulation including “cytokine–cytokine receptor interaction”, “cell adhesion molecules”, “complement and coagulation cascade” and “PI3K-AKT signalling pathway” (Fig. 4E). Taken together, these data show that ex vivo expansion of MSCs at high cell confluency can enhance properties associated with immunosuppression suggesting our approach is a generalizable and non-invasive strategy for enriching MSCs for enhanced immunomodulatory functions (Fig. 4F).

### MSC_HC_ reduces inflammation more than MSC_LC_ in human PBMC-induced mouse GVHD model

**Fig. 5:**
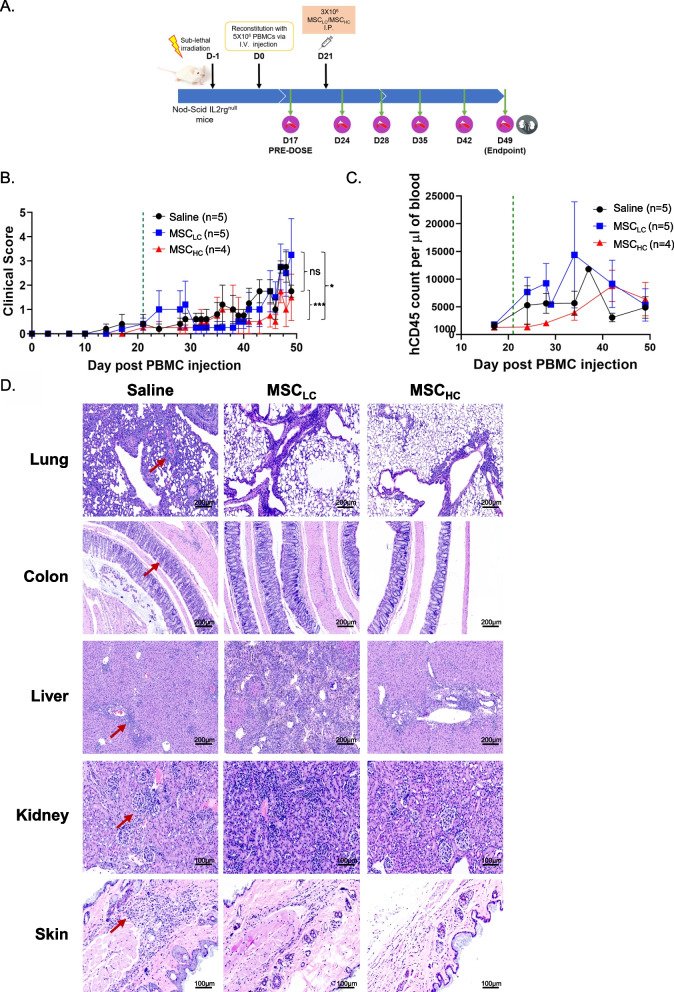
MSC_HC_ reduces inflammation and disease severity in a graft-versus-host disease (GVHD) mouse model in vivo. **A** Timeline of treatment regimen of PBMC injection and MSCs administration. **B** Clinical scoring of GVHD-bearing mice with saline (*n* = 5), MSC_LC_ (*n* = 5) and MSC_HC_ (*n* = 4) with MSC administration from day 21 post-PBMC injection as indicated by green line. **C** Human CD45 blood count in treatment groups. **D** Representative histology images of lung, colon, liver, kidney and skin from each treatment group. The presence of immune cell infiltrates indicated by red arrows. Wilcoxon matched-pairs signed-rank test was performed between selected pairs. *p ≤ 0.05; *** *p* ≤ 0.001; ns represents non-significant. There were originally 15 mice for the study, but human CD45 engraftment was not successful in one mouse on Day 17 post-PBMC injection, hence to be removed from the study

To examine whether MSC_HC_ exerts greater immunosuppression than MSC_LC_ in vivo, we investigated in a humanized mouse model of graft-versus-host disease (GVHD). Briefly, NOD-SCID IL2rg^null^ (NSG) female mice were irradiated and injected with 5 x 10^6^ human PBMCs via tail vein. We first performed a pre-dose blood withdrawal at 17 days post-PBMC injection to confirm successful immune reconstitution of human CD45^+^ (hCD45) cells in these GVHD-bearing mice before we administered human MSC_LC_, MSC_HC_ or saline. We observed that the levels of human hCD45 cells per µl blood was in the range of 222.8 to 3215.8, with an average of 1559.4 across all PBMC-engrafted mice. This provided us an opportunity to evaluate the therapeutic effect of MSCs from the reduction in hCD45 cell count in response to MSC administration. Expanded MSCs at passage 4 were administered via intraperitoneal route into the mice 21 days post-PBMC injection. GVHD symptoms were monitored daily by observing the mice and by weekly blood withdrawal (Fig. 5A). Based on a standard clinical scoring system [59] to determine GVHD severity (Additional file 5: Fig. S5), mice administered with MSC_HC_ had reduced body weight loss (< 10%) and experienced normal or mild decrease in movement as compared to MSC_LC_ or saline-treated GVHD mice (Fig. 5B). The presence of hCD45 cells in blood samples indicates the extent of engraftment in the human PBMC-induced mouse GVHD model. We observed one mouse death each, in MSC_LC_-treated (Day 29) and saline-treated (Day 37) groups while all PBMC-engrafted mice treated with MSC_HC_ cells survived until the analysis endpoint (Day 49). We found that hCD45 cells were overall lower in MSC_HC_-treated GVHD mice (n = 4), as compared to saline or MSC_LC_ treatment groups (n = 5 and n = 5, respectively) up to 14 days after MSC administration (35 days post-PBMC injection), suggesting an initial robust effect (Fig. 5C, Additional file 6: Fig. S6). Analyses of hCD45 blood count analysis revealed that single dose of MSC_HC_ can suppress or delay T cell proliferation by ~ 2.6–4.4-fold for 7–14 days compared to PBMC-engrafted mice treated with MSC_LC_ or saline. After 14 days post-MSC_HC_ treatment, we observed that the immunosuppressive effect of MSC_HC_ began to decline as the suppression on hCD45 cell count was not observed. Nevertheless, we observed reduced lymphocyte infiltration and improved tissue integrity in MSC_HC_-treated GVHD mice as compared to MSC_LC_ based on histological evaluation of the lungs, colon, liver, kidney and skin and consistent with improved survival (Fig. 5D). The onset of GVHD is triggered by human T cells from the graft that recognizes the cells of the recipient host as foreign. From histological assessment, mice treated with MSC_LC_ and saline suffered most damage in most organs such as lungs, colon, liver, kidneys and skin, which is characterized by thickening of bronchial wall, colonic muscle wall and massive infiltration of immune cells. Thus, our results indicate that even a single dose of MSC_HC_ can decrease inflammation and tissue damage in humanized GVHD mouse model, opening the door for further investigations of MSCs as a treatment for inflammatory diseases.

## Discussion

MSCs can regulate the functions of immune cells both in vitro and in vivo; however, their clinical efficacy as an immunotherapy has been inconsistent [60]. Donor heterogeneity, cell source [61] and a lack of standardized isolation and expansion protocols have posed challenges for the use of MSCs as a cell therapy. Preconditioning MSCs with pro-inflammatory cytokines or other priming approaches to improve their immunomodulatory functions have been reported [30, 62–64]. However, priming MSCs could introduce more variability during expansion and the impact of MSC culture conditions on specific immune cell populations has not been fully investigated. Moreover, translating primed MSCs to the clinic has significant limitations due to the high cost of producing these cells as well as safety concerns related to tumorigenic potential [30]. Thus, we focused on identifying conditions and mechanistic underpinnings that lead to enhanced immunomodulatory functions of basal MSCs in the context of T cell proliferation in the absence of cytokine priming.

Our study demonstrates that harvesting MSCs at high density enhanced the ability of MSCs to suppress proliferation of activated T cells in 4 out of 6 donors. Consequently, we measured levels of pro-inflammatory cytokines (IFN-ɣ, IL-17A) which were decreased in co-cultures with MSC_HC_ donors that showed the most robust effects on T cell suppression. Prior studies have demonstrated that metabolic remodelling towards enhanced glycolysis correlates with MSCs that are immunosuppressive [47, 51, 52], accompanied with an increased lactate production that is known to suppress immune cell function [65, 66] including T cell proliferation [54, 55]. Notably, increased lactate production strongly correlated with the same MSC_HC_ donors that showed enhanced immunomodulation and cytokine expression. Although lactate is considered a glycolytic by-product, it is now being recognized as a signalling molecule involved in cell survival [67, 68] which could be critical for their enhanced activity in vivo. We also observed a trend for metabolic reprogramming towards glycolysis in MSC_HC_ for most donors using the Seahorse assay. Thus, monitoring IFN-ɣ and IL-17A as well as lactate levels during MSC expansion could be used as critical quality attributes for identifying MSCs with an enhanced ability to suppress T cell proliferation.

Although several transcriptomic studies have shown that genes involved in immunomodulation are highly expressed in human MSCs cultured at high confluency as compared to low confluency [6, 32, 37], these studies are limited by the number of donors tested, making it difficult to identify pathways that robustly correlate with immunomodulatory activity. By performing a comprehensive transcriptome analysis across six donors, we identified several differentially regulated genes in MSC_HC_ with known roles in immunomodulation including *TRIM29 *[69], *BPIFB4 *[70], *MMP3 *[71], *SPP1 *[72], which could hence explain enhanced immunomodulatory properties in MSC_HC_ compared to MSC_LC_. By further comparing gene expression patterns in responders MSC_HC_ (Donor 1, 2, 4 and 5) which exhibit enhanced immunosuppression towards T cell proliferation compared to non-responders (Donor 3 and 6), we observed an enrichment of pathways regulating immune response including cytokine–cytokine receptor interaction pathway, cell adhesion pathway and PI3K-AKT signalling pathway. Within the cytokine–cytokine receptor interaction pathway, genes associated with MSC-mediated immunomodulation such as *BMP4 *[73], *CCL26 *[74], *CX3CL1 *[75, 76], *IL6 *[77], *LEP *[78] and *TGFBR1 *[79] were identified. We also observed the differential expression of SDC1 [80], which was highlighted in the cell adhesion pathway that mediates interaction between MSCs and immune cells. It is known that the activation of PI3K-AKT signalling pathway confers anti-inflammatory functions to MSCs [81], which suggests that its enrichment in the responders may explain the enhanced immunosuppression exhibited by MSC_HC_. Overall, the ability to control expression of these genes to enhance MSC-mediated immunomodulation functions provides an exciting area to develop approaches to overcome the challenges of MSC therapy, which are donor heterogeneity and variations to MSC properties during ex vivo expansion.

Steroid-based anti-inflammatory therapy is often used as first line therapy, but developing resistance to steroids is frequently observed in patients and lead to high mortality [14, 19, 23]. The therapeutic potential of MSCs offers an alternative treatment for steroid-refractory patients. The ability to define culture conditions that enrich for MSCs with enhanced immunomodulatory activity could facilitate their use as a treatment of diseases characterized by pro-inflammatory T cells like GVHD [82]. Collectively, our in vivo data support our in vitro findings demonstrating that MSC_HC_ can suppress T cell proliferation in peripheral blood. Administration of even a single dose at Day 21 significantly reduced the GVHD clinical score, prolonged survival, reduced immune cell infiltration to organs and organs damage compared to MSC_LC_ and saline (non-MSC)-treated mice. Thus, results from the peripheral blood count, GVHD clinical score and histopathological analyses support that MSC_HC_ suppresses T cell proliferation and inflammation in a humanized murine models of xenogeneic GVHD.

Although the immunosuppressive effect of MSC_HC_ cells began to wane after 14 days post-MSC_HC_ treatment, we observed a significant decrease in lymphocyte infiltration in the organs and improved survival. Thus, it is possible that multiple dosing of MSC_HC_ cells to PBMC-engrafted mice every 14 days or increasing the number of MSC_HC_ injected could prolong the improvement of GVHD clinical score.

## Conclusion

The potential of MSCs in cell therapy is often limited by donor heterogeneity and differences in cell culture protocols across laboratories. Taken together, we show that enrichment of immunosuppressive functions during ex vivo expansion of MSCs can be achieved by culturing at high confluency. Future studies of MSC_HC_ as a treatment of steroid-resistant GVHD or whether a maximal effect can be achieved in combination with existing drugs such as prednisone and ibrutinib [83] warrants further investigation that could greatly impact the lives of many patients.

### Supplementary Information

**Additional file 1: Fig. S1.** Representative microscopy images of low confluency (MSC_LC_) and high confluency (MSC_HC_) for Donor 2 to 6. Scale bars at 1 mm with confluency values as indicated.

**Additional file 2: Fig. S2.** MSC multi-lineage differentiation remains similar in different culture confluency conditions. (A) In vitro differentiation of MSC_LC_ and MSC_HC_ from Donor 2 to Donor 6 (Scale bars:100μm). Alizarin red staining for calcium deposits detected in osteoblasts, Oil red O staining for oil droplets in adipocytes and collagen II staining was performed for chondrocytes. (B) Differentiation assays performed in technical triplicates and quantified for Donor 2 to Donor 6 as compared to undifferentiated cells (UD). One-way ANOVA (Tukey's multiple comparison test) or unpaired t-test (two-tailed) was performed as statistical test. *p ≤0.05; ** p ≤0.01 ***p ≤0.001; ns represents non-significant.

**Additional file 3: Fig. S3.** Flow cytometry-mediated characterization of surface markers in each donor at Passage 2 before culture expansion. BM-MSCs at Passage 2 were positive for MSC markers CD73, CD90, CD105, but were negative (<5%) for CD34 and CD45.

**Additional file 4: Fig. S4.** Preparation of CD3^+^ T cells for in vitro co-culture assays. (A) CD3^+^ isolation using negative immunoselection was performed using human peripheral blood mononuclear cells and >90% purity was achieved after selection. (B) Flow cytometric analysis of CD4^+^ T cells and CD8^+^ T cells from PBMCs of a healthy donor.

**Additional file 5: Fig. S5.** Mouse GVHD clinical scoring system. Assessment of disease severity in mice based on fur texture, movements, posture, body weight loss and skin integrity.

**Additional file 6: Fig. S6.** Human CD45 (hCD45) blood cell count in GVHD mice at indicated day post-PBMC injection. hCD45 blood cell count in µl of blood for GVHD-bearing mice with saline (n=5), MSCLC (n=5) and MSCHC (n=4) on Day 17, 24, 28, 35, 42 post-PBMC injection.

**Additional file 7: Supplemental Table 1.** Differentially expressed genes (DEGs) in bone marrow-derived MSCs from six human donors, cultured at high confluency (MSC-HC) versus low confluency (MSC-LC).

**Additional file 8: Supplemental Table 2.** Differentially expressed genes (DEGs) in bone marrow-derived MSCs unique to responders (Donor 1, Donor 2, Donor 4, Donor 5), cultured at high confluency (MSC-HC) versus low confluency (MSC-LC).

## Data Availability

RNA-seq data deposited in the Gene Expression Omnibus (GEO) database are publicly available under accession number GSE231354 at the following link: https://www.ncbi.nlm.nih.gov/geo/query/acc.cgi?&acc=GSE231354.

## Abbreviations

APC: Allophycocyanin
BV: Brilliant violet
CTV: Celltrace violet
DEGs: Differentially expressed genes
DMEM: Dulbecco’s modified Eagle medium
ECAR: Extracellular acidification rate
ELISA: Enzyme-linked immunosorbent assay
FBS: Foetal bovine serum
FC: Fold change
FITC: Fluorescein isothiocyanate
FI_nd_: Peak fluorescence intensity of the viable non-divided cells
FMO: Fluorescence-minus-one
GVHD: Graft-versus-host disease
hCD45: Human CD45^+^ cells
ISCT: International Society of Cell and Gene Therapy
MSC: Mesenchymal stromal cell
MSC_HC_: Mesenchymal stromal cells cultured at high confluency
MSC_LC_: Mesenchymal stromal cells cultured at low confluency
MFI_all_: Median fluorescence intensity of all viable T cells
NSG: NOD-SCID IL2rg^null^ mice
PBMC: Peripheral blood mononuclear cells
OD: Optical densities
PBS: Phosphate-buffered saline
PCA: Principle component analysis
PE: Phycoerythrin
PerCP: Peridinin chlorophyll protein
PHA: Phytohaemagglutin A
PI: Proliferative index
RBC: Red blood cells
sGAGs: Glycosaminoglycans

## References

[1] Mackenzie TC, Flake AW (2001). Multilineage differentiation of human MSC after in utero transplantation. Cytotherapy.

[2] Baghaei K, Hashemi SM, Tokhanbigli S, Asadi Rad A, Assadzadeh-Aghdaei H, Sharifian A (2017). Isolation, differentiation, and characterization of mesenchymal stem cells from human bone marrow. Gastroenterol Hepatol Bed Bench.

[3] Pak J, Lee JH, Lee SH (2014). Regenerative repair of damaged meniscus with autologous adipose tissue-derived stem cells. Biomed Res Int.

[4] Choi YS, Park Y-B, Ha C-W, Kim JA, Heo J-C, Han W-J (2017). Different characteristics of mesenchymal stem cells isolated from different layers of full term placenta. PLoS ONE.

[5] Mennan C, Wright K, Bhattacharjee A, Balain B, Richardson J, Roberts S (2013). Isolation and characterisation of mesenchymal stem cells from different regions of the human umbilical cord. Biomed Res Int.

[6] Lee MW, Kim DS, Yoo KH, Kim HR, Jang IK, Lee JH (2013). Human bone marrow-derived mesenchymal stem cell gene expression patterns vary with culture conditions. Blood Res.

[7] Poon Z, Lee WC, Guan G, Nyan LM, Lim CT, Han J (2015). Bone marrow regeneration promoted by biophysically sorted osteoprogenitors from mesenchymal stromal cells. Stem Cells Transl Med.

[8] Kim DS, Lee MW, Lee T-H, Sung KW, Koo HH, Yoo KH (2017). Cell culture density affects the stemness gene expression of adipose tissue-derived mesenchymal stem cells. Biomed Rep.

[9] Carrión F, Nova E, Luz P, Apablaza F, Figueroa F (2011). Opposing effect of mesenchymal stem cells on Th1 and Th17 cell polarization according to the state of CD4+ T cell activation. Immunol Lett.

[10] Cho KS, Park HK, Park HY, Jung JS, Jeon SG, Kim YK (2009). IFATS collection: Immunomodulatory effects of adipose tissue-derived stem cells in an allergic rhinitis mouse model. Stem Cells.

[11] Di Nicola M, Carlo-Stella C, Magni M, Milanesi M, Longoni PD, Matteucci P (2002). Human bone marrow stromal cells suppress T-lymphocyte proliferation induced by cellular or nonspecific mitogenic stimuli. Blood.

[12] Djouad F, Charbonnier LM, Bouffi C, Louis-Plence P, Bony C, Apparailly F (2007). Mesenchymal stem cells inhibit the differentiation of dendritic cells through an interleukin-6-dependent mechanism. Stem Cells.

[13] Jiang W, Xu J (2020). Immune modulation by mesenchymal stem cells. Cell Prolif.

[14] Kuçi Z, Bönig H, Kreyenberg H, Bunos M, Jauch A, Janssen JW (2016). Mesenchymal stromal cells from pooled mononuclear cells of multiple bone marrow donors as rescue therapy in pediatric severe steroid-refractory graft-versus-host disease: a multicenter survey. Haematologica.

[15] Huang P, Gebhart N, Richelson E, Brott TG, Meschia JF, Zubair AC (2014). Mechanism of mesenchymal stem cell-induced neuron recovery and anti-inflammation. Cytotherapy.

[16] Rasmusson I (2006). Immune modulation by mesenchymal stem cells. Exp Cell Res.

[17] Yang H, Sun J, Li Y, Duan WM, Bi J, Qu T (2016). Human umbilical cord-derived mesenchymal stem cells suppress proliferation of PHA-activated lymphocytes in vitro by inducing CD4(+)CD25(high)CD45RA(+) regulatory T cell production and modulating cytokine secretion. Cell Immunol.

[18] Ramasamy R, Tong CK, Seow HF, Vidyadaran S, Dazzi F (2008). The immunosuppressive effects of human bone marrow-derived mesenchymal stem cells target T cell proliferation but not its effector function. Cell Immunol.

[19] Kebriaei P, Hayes J, Daly A, Uberti J, Marks DI, Soiffer R (2020). A phase 3 randomized study of remestemcel-L versus placebo added to second-line therapy in patients with steroid-refractory acute graft-versus-host disease. Biol Blood Marrow Transpl.

[20] Staines R. Shock as FDA rejects Mesoblast’s Ryoncil in paediatric graft versus host disease. 2020. https://pharmaphorum.com/news/fda-issues-shock-rejection-for-mesoblasts-ryoncil-in-graft-versus-host-disease/.

[21] Le Blanc K, Rasmusson I, Sundberg B, Götherström C, Hassan M, Uzunel M (2004). Treatment of severe acute graft-versus-host disease with third party haploidentical mesenchymal stem cells. Lancet (London, England).

[22] Le Blanc K, Frassoni F, Ball L, Locatelli F, Roelofs H, Lewis I (2008). Mesenchymal stem cells for treatment of steroid-resistant, severe, acute graft-versus-host disease: a phase II study. Lancet (London, England).

[23] Cope AP (2008). T cells in rheumatoid arthritis. Arthritis Res Ther.

[24] Skapenko A, Leipe J, Lipsky PE, Schulze-Koops H (2005). The role of the T cell in autoimmune inflammation. Arthritis Res Ther.

[25] Prasanna SJ, Gopalakrishnan D, Shankar SR, Vasandan AB (2010). Pro-inflammatory cytokines, IFNgamma and TNFalpha, influence immune properties of human bone marrow and Wharton jelly mesenchymal stem cells differentially. PLoS ONE.

[26] Chinnadurai R, Copland IB, Patel SR, Galipeau J (2014). IDO-independent suppression of T cell effector function by IFN-γ-licensed human mesenchymal stromal cells. J Immunol.

[27] Noone C, Kihm A, English K, O'Dea S, Mahon BP (2013). IFN-γ stimulated human umbilical-tissue-derived cells potently suppress NK activation and resist NK-mediated cytotoxicity in vitro. Stem Cells Dev.

[28] Mennan C, Brown S, McCarthy H, Mavrogonatou E, Kletsas D, Garcia J (2016). Mesenchymal stromal cells derived from whole human umbilical cord exhibit similar properties to those derived from Wharton's jelly and bone marrow. FEBS Open Bio.

[29] English K, Barry FP, Field-Corbett CP, Mahon BP (2007). IFN-gamma and TNF-alpha differentially regulate immunomodulation by murine mesenchymal stem cells. Immunol Lett.

[30] Noronha NdC, Mizukami A, Caliári-Oliveira C C, Cominal JG, Rocha JLM, Covas DT (2007). Priming approaches to improve the efficacy of mesenchymal stromal cell-based therapies. Stem Cell Res.

[31] Herzig MC, Christy BA, Montgomery RK, Delavan CP, Jensen KJ, Lovelace SE (2021). Interactions of human mesenchymal stromal cells with peripheral blood mononuclear cells in a Mitogenic proliferation assay. J Immunol Methods.

[32] Lee MW, Kim DS, Ryu S, Jang IK, Kim HJ, Yang JM (2013). Effect of ex vivo culture conditions on immunosuppression by human mesenchymal stem cells. Biomed Res Int.

[33] Krampera M, Galipeau J, Shi Y, Tarte K, Sensebe L (2013). Immunological characterization of multipotent mesenchymal stromal cells—the International Society for Cellular Therapy (ISCT) working proposal. Cytotherapy.

[34] Malard F, Huang X-J, Sim JPY (2020). Treatment and unmet needs in steroid-refractory acute graft-versus-host disease. Leukemia.

[35] Resnick IB, Barkats C, Shapira MY, Stepensky P, Bloom AI, Shimoni A (2013). Treatment of severe steroid resistant acute GVHD with mesenchymal stromal cells (MSC). Am J Blood Res.

[36] Markus B, Annette E. Flow cytometric analysis of in vitro proliferation and immunosuppression by fluorescent dyes that are successively dispersed upon cell division 2007. http://www.cyto.purdue.edu/cdroms/cyto10a/educationandresearch/flowanalysis.html.

[37] Kim DS, Lee MW, Yoo KH, Lee TH, Kim HJ, Jang IK (2014). Gene expression profiles of human adipose tissue-derived mesenchymal stem cells are modified by cell culture density. PLoS ONE.

[38] Sekiya I, Larson BL, Smith JR, Pochampally R, Cui JG, Prockop DJ (2002). Expansion of human adult stem cells from bone marrow stroma: conditions that maximize the yields of early progenitors and evaluate their quality. Stem Cells.

[39] Pavel M, Renna M, Park SJ, Menzies FM, Ricketts T, Füllgrabe J (2018). Contact inhibition controls cell survival and proliferation via YAP/TAZ-autophagy axis. Nat Commun.

[40] Dominici M, Le Blanc K, Mueller I, Slaper-Cortenbach I, Marini F, Krause D (2006). Minimal criteria for defining multipotent mesenchymal stromal cells. The International Society for Cellular Therapy position statement. Cytotherapy.

[41] Kaiser S, Hackanson B, Follo M, Mehlhorn A, Geiger K, Ihorst G (2007). BM cells giving rise to MSC in culture have a heterogeneous CD34 and CD45 phenotype. Cytotherapy.

[42] Technologies S. MesenCult™ osteogenic differentiation kit (human). https://www.stemcell.com/products/mesencult-osteogenic-differentiation-kit-human.html#section-protocols-and-documentation.

[43] Technologies S. MesenCult™ adipogenic differentiation kit (human). https://www.stemcell.com/products/mesencult-adipogenic-differentiation-medium-human.html.

[44] Technologies S. MesenCult™-ACF chondrogenic differentiation kit. https://www.stemcell.com/mesencult-acf-chondrogenic-differentiation-medium.html.

[45] Huse M (2009). The T-cell-receptor signaling network. J Cell Sci.

[46] Grégoire C, Ritacco C, Hannon M, Seidel L, Delens L, Belle L (2019). Comparison of mesenchymal stromal cells from different origins for the treatment of graft-vs.-host-disease in a humanized mouse model. Front Immunol.

[47] Liu Y, Yuan X, Muñoz N, Logan TM, Ma T (2019). Commitment to aerobic glycolysis sustains immunosuppression of human mesenchymal stem cells. Stem Cells Transl Med.

[48] Palomares Cabeza V, Hoogduijn MJ, Kraaijeveld R, Franquesa M, Witte-Bouma J, Wolvius EB (2019). Pediatric mesenchymal stem cells exhibit immunomodulatory properties toward allogeneic t and b cells under inflammatory conditions. Front Bioeng Biotechnol.

[49] Hess NJ, Hudson AW, Hematti P, Gumperz JE (2020). Early T cell activation metrics predict graft-versus-host disease in a humanized mouse model of hematopoietic stem cell transplantation. J Immunol.

[50] Nervi B, Rettig MP, Ritchey JK, Wang HL, Bauer G, Walker J (2007). Factors affecting human T cell engraftment, trafficking, and associated xenogeneic graft-vs-host disease in NOD/SCID beta2mnull mice. Exp Hematol.

[51] Contreras-Lopez R, Elizondo-Vega R, Luque-Campos N, Torres MJ, Pradenas C, Tejedor G (2021). The ATP synthase inhibition induces an AMPK-dependent glycolytic switch of mesenchymal stem cells that enhances their immunotherapeutic potential. Theranostics.

[52] Contreras-Lopez RA, Elizondo-Vega R, Torres MJ, Vega-Letter AM, Luque-Campos N, Paredes-Martinez MJ (2020). PPARβ/δ-dependent MSC metabolism determines their immunoregulatory properties. Sci Rep.

[53] Lee YK, Lim JJ, Jeoun UW, Min S, Lee EB, Kwon SM (2017). Lactate-mediated mitoribosomal defects impair mitochondrial oxidative phosphorylation and promote hepatoma cell invasiveness. J Biol Chem.

[54] Rostamian H, Khakpoor-Koosheh M, Jafarzadeh L, Masoumi E, Fallah-Mehrjardi K, Tavassolifar MJ (2022). Restricting tumor lactic acid metabolism using dichloroacetate improves T cell functions. BMC Cancer.

[55] Quinn WJ, Jiao J, TeSlaa T, Stadanlick J, Wang Z, Wang L (2020). Lactate limits T cell proliferation via the NAD(H) redox state. Cell Rep.

[56] Wobma HM, Kanai M, Ma SP, Shih Y, Li HW, Duran-Struuck R (2018). Dual IFN-γ/hypoxia priming enhances immunosuppression of mesenchymal stromal cells through regulatory proteins and metabolic mechanisms. J Immunol Regener Med.

[57] Liu J, Hao H, Xia L, Ti D, Huang H, Dong L (2015). Hypoxia pretreatment of bone marrow mesenchymal stem cells facilitates angiogenesis by improving the function of endothelial cells in diabetic rats with lower ischemia. PLoS ONE.

[58] Schmidt CA, Fisher-Wellman KH, Neufer PD (2021). From OCR and ECAR to energy: perspectives on the design and interpretation of bioenergetics studies. J Biol Chem.

[59] Lai HY, Chou TY, Tzeng CH, Lee OK (2012). Cytokine profiles in various graft-versus-host disease target organs following hematopoietic stem cell transplantation. Cell Transpl.

[60] Kaundal U, Bagai U, Rakha A (2018). Immunomodulatory plasticity of mesenchymal stem cells: a potential key to successful solid organ transplantation. J Transl Med.

[61] Via AG, Frizziero A, Oliva F (2012). Biological properties of mesenchymal stem cells from different sources. Muscles Ligaments Tendons J.

[62] Baldari S AG, Di Rocco G A, Piccoli M F (2017). Challenges and strategies for improving the regenerative effects of mesenchymal stromal cell-based therapies. Int J Mol Sci.

[63] Hu C, Li L (2018). Preconditioning influences mesenchymal stem cell properties in vitro and in vivo. J Cell Mol Med.

[64] Najar M, Krayem M, Merimi M, Burny A, Meuleman N, Bron D (2018). Insights into inflammatory priming of mesenchymal stromal cells: functional biological impacts. Inflamm Res.

[65] Brand A, Singer K, Koehl GE, Kolitzus M, Schoenhammer G, Thiel A (2016). LDHA-associated lactic acid production blunts tumor immunosurveillance by T and NK Cells. Cell Metab.

[66] Selleri S, Bifsha P, Civini S, Pacelli C, Dieng M, Lemieux W (2014). Human mesenchymal stromal cell-secreted lactate induces M2-macrophage differentiation by metabolic reprogramming. Oncotarget.

[67] Taddei ML, Pietrovito L, Leo A, Chiarugi P (2020). Lactate in sarcoma microenvironment: much more than just a waste product. Cells.

[68] Tauffenberger A, Fiumelli H, Almustafa S, Magistretti PJ (2019). Lactate and pyruvate promote oxidative stress resistance through hormetic ROS signaling. Cell Death Dis.

[69] Xing J, Zhang A, Minze LJ, Li XC, Zhang Z (2018). TRIM29 negatively regulates the type I IFN production in response to RNA virus. J Immunol.

[70] Ciaglia E, Montella F, Lopardo V, Ferrario A, Cattaneo M (2020). Circulating BPIFB4 levels associate with and influence the abundance of reparative monocytes and macrophages in long living individuals. Front Immunol.

[71] Wan J, Zhang G, Li X, Qiu X, Ouyang J, Dai J (2021). Matrix metalloproteinase 3: a promoting and destabilizing factor in the pathogenesis of disease and cell differentiation. Front Physiol.

[72] Weber GF, Zawaideh S, Hikita S, Kumar VA, Cantor H, Ashkar S (2002). Phosphorylation-dependent interaction of osteopontin with its receptors regulates macrophage migration and activation. J Leukoc Biol.

[73] Hu L, Xu J, Wu T, Fan Z, Sun L, Liu Y (2020). Depletion of ID3 enhances mesenchymal stem cells therapy by targeting BMP4 in Sjögren’s syndrome. Cell Death Dis.

[74] Dong Z, Cao L, Guo L, Hong Y, Cao J, Chen X (2020). CCL26 regulates the proportion of CD4+ CD25+ FOXP3+ Tregs and the production of inflammatory factors in peripheral blood mononuclear cells following acute ischemic stroke via the STAT5 pathway [Retracted]. Exp Ther Med.

[75] Huang L, Xu W, Xu G (2013). Transplantation of CX3CL1-expressing mesenchymal stem cells provides neuroprotective and immunomodulatory effects in a rat model of retinal degeneration. Ocul Immunol Inflamm.

[76] Giunti D, Parodi B, Usai C, Vergani L, Casazza S, Bruzzone S (2012). Mesenchymal stem cells shape microglia effector functions through the release of CX3CL1. Stem Cells.

[77] Philipp D, Suhr L, Wahlers T, Choi YH, Paunel-Görgülü A (2018). Preconditioning of bone marrow-derived mesenchymal stem cells highly strengthens their potential to promote IL-6-dependent M2b polarization. Stem Cell Res Ther.

[78] Otero M, Lago R, Gomez R, Dieguez C, Lago F, Gómez-Reino J (2006). Towards a pro-inflammatory and immunomodulatory emerging role of leptin. Rheumatology.

[79] Liu L, Cheng M, Zhang T, Chen Y, Wu Y, Wang Q (2022). Mesenchymal stem cell-derived extracellular vesicles prevent glioma by blocking M2 polarization of macrophages through a miR-744-5p/TGFB1-dependent mechanism. Cell Biol Toxicol.

[80] Jones FK, Stefan A, Kay AG, Hyland M, Morgan R, Forsyth NR (2020). Syndecan-3 regulates MSC adhesion, ERK and AKT signalling in vitro and its deletion enhances MSC efficacy in a model of inflammatory arthritis in vivo. Sci Rep.

[81] Xu C, Feng C, Huang P, Li Y, Liu R, Liu C (2022). TNFα and IFNγ rapidly activate PI3K-AKT signaling to drive glycolysis that confers mesenchymal stem cells enhanced anti-inflammatory property. Stem Cell Res Ther.

[82] Wang L-T, Ting C-H, Yen M-L, Liu K-J, Sytwu H-K, Wu KK (2016). Human mesenchymal stem cells (MSCs) for treatment towards immune- and inflammation-mediated diseases: review of current clinical trials. J Biomed Sci.

[83] Jaglowski SM, Blazar BR (2018). How ibrutinib, a B-cell malignancy drug, became an FDA-approved second-line therapy for steroid-resistant chronic GVHD. Blood Adv.

