# Supplementary figures and images for: Expansion of human bone marrow-derived mesenchymal stromal cells with enhanced immunomodulatory properties
Source: Stem Cell Res Ther. 2023 Sep 19;14:259. doi: 10.1186/s13287-023-03481-7 (PMC10510228; doi:10.1186/s13287-023-03481-7)

# Supplementary Figure 1

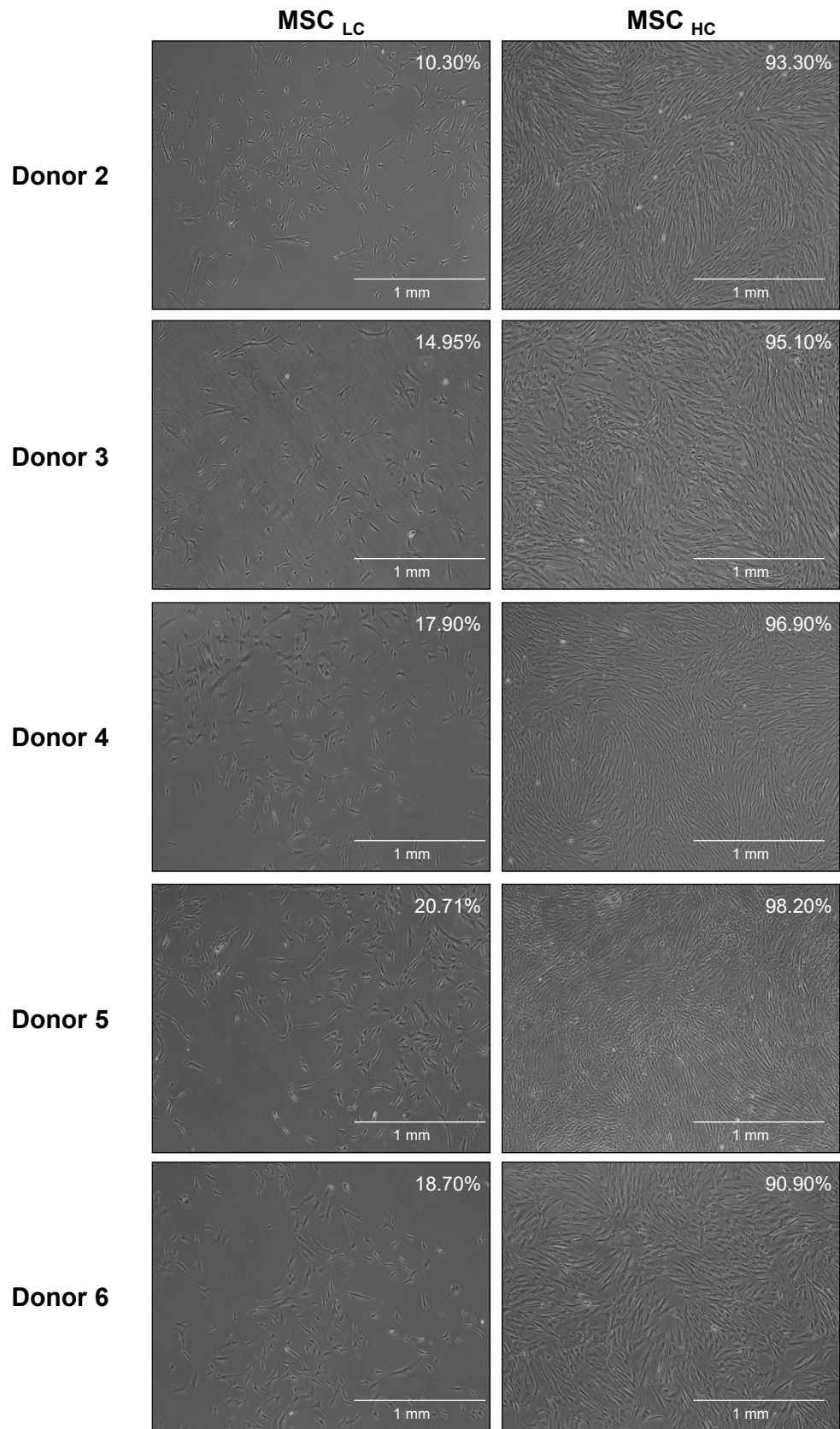

Supplement: Supplementary file 1 — Additional file 1: Fig. S1. Representative microscopy images of low confluency (MSCLC) and high confluency (MSCHC) for Donor 2 to 6. Scale bars at 1 mm with confluency values as indicated. [file 13287_2023_3481_MOESM1_ESM.pdf]

# Supplementary Figure 3

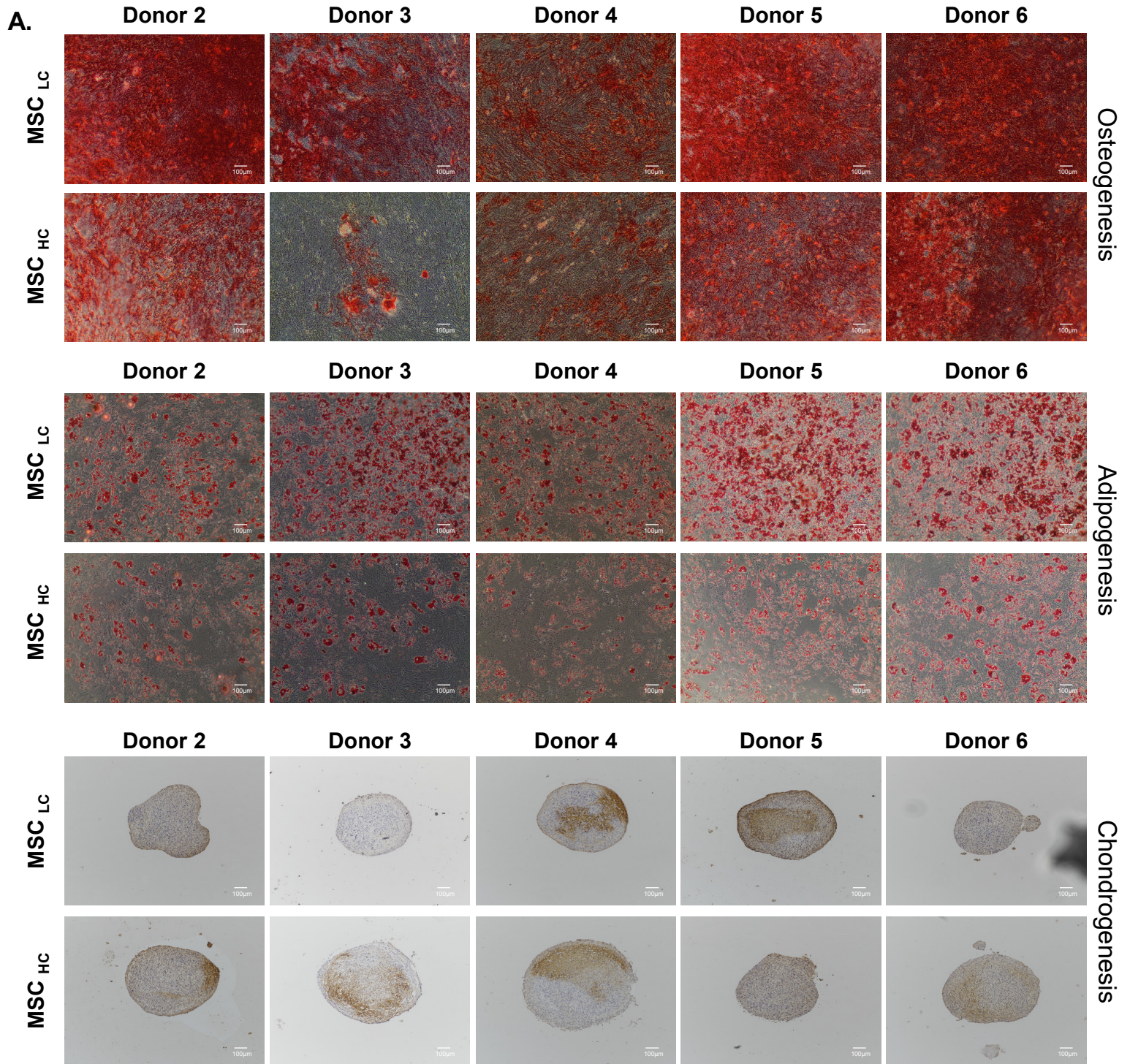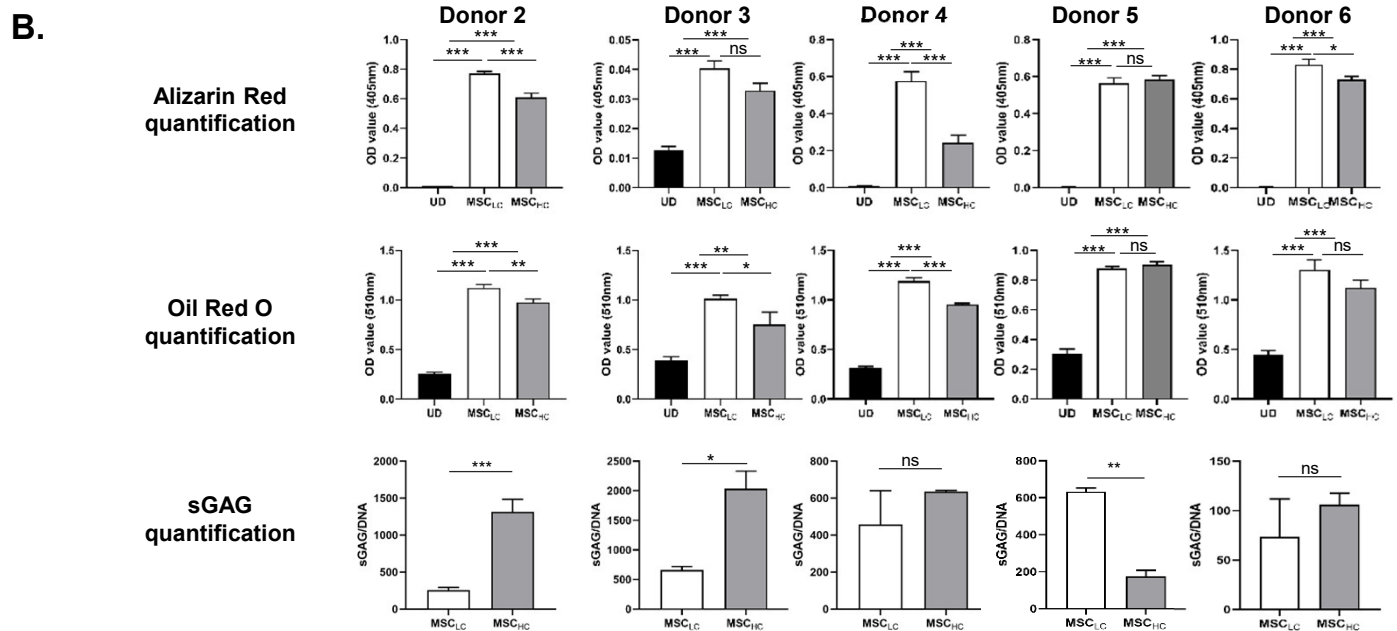

Supplement: Supplementary file 3 — Additional file 3: Fig. S3. Flow cytometry-mediated characterization of surface markers in each donor at Passage 2 before culture expansion. BM-MSCs at Passage 2 were positive for MSC markers CD73, CD90, CD105, but were negative (<5%) for CD34 and CD45. [file 13287_2023_3481_MOESM3_ESM.pdf]

## Supplementary Figure 4

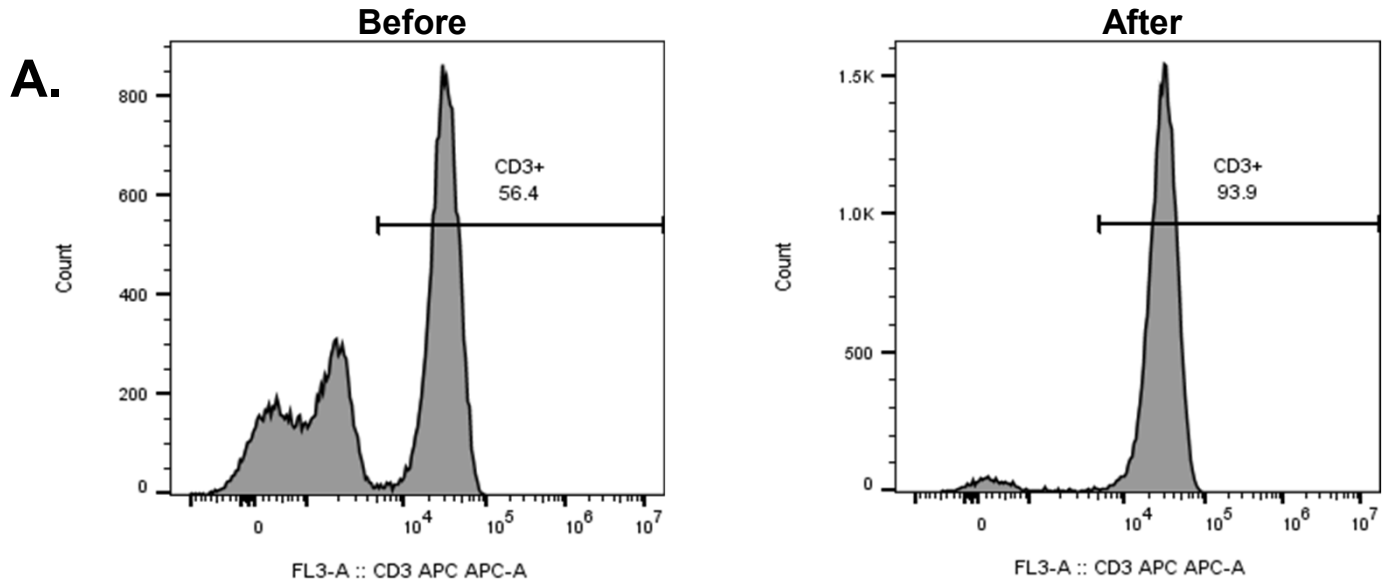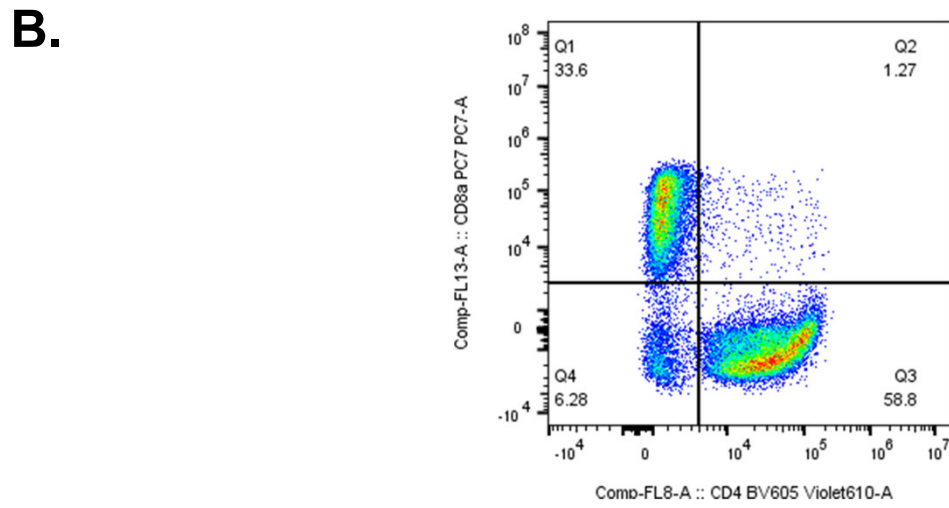

Supplement: Supplementary file 4 — Additional file 4: Fig. S4. Preparation of CD3+ T cells for in vitro co-culture assays. (A) CD3+ isolation using negative immunoselection was performed using human peripheral blood mononuclear cells and >90% purity was achieved after selection. (B) Flow cytometric analysis of CD4+ T cells and CD8+ T cells from PBMCs of a healthy donor. [file 13287_2023_3481_MOESM4_ESM.pdf]
