# Supplementary material for: Expansion of human bone marrow-derived mesenchymal stromal cells with enhanced immunomodulatory properties
Source: Stem Cell Res Ther. 2023 Sep 19;14:259. doi: 10.1186/s13287-023-03481-7 (PMC10510228; doi:10.1186/s13287-023-03481-7)
Supplement: Supplementary file 2 — Additional file 2: Fig. S2. MSC multi-lineage differentiation remains similar in different culture confluency conditions. (A) In vitro differentiation of MSCLC and MSCHC from Donor 2 to Donor 6 (Scale bars:100μm). Alizarin red staining for calcium deposits detected in osteoblasts, Oil red O staining for oil droplets in adipocytes and collagen II staining was performed for chondrocytes. (B) Differentiation assays performed in technical triplicates and quantified for Donor 2 to Donor 6 as compared to undifferentiated cells (UD). One-way ANOVA (Tukey's multiple comparison test) or unpaired t-test (two-tailed) was performed as statistical test. *p ≤0.05; ** p ≤0.01 ***p ≤0.001; ns represents non-significant. [file 13287_2023_3481_MOESM2_ESM.pdf]

Supplementary Figure 2

	% positive cells				
**Donor**	**CD34**	**CD45**	**CD73**	**CD90**	**CD105**
1	0.16	0.02	99.6	99.9	99.9
2	0.13	0.09	99.6	99.8	99.6
3	0.54	0.03	96.9	99.8	99.8
4	0.51	0.04	98	99.8	99.8
5	0.4	0.01	97.2	99.4	99.9
6	0.25	0.37	97.4	99.9	99.8
