# Supplementary material for: Expansion of human bone marrow-derived mesenchymal stromal cells with enhanced immunomodulatory properties
Source: Stem Cell Res Ther. 2023 Sep 19;14:259. doi: 10.1186/s13287-023-03481-7 (PMC10510228; doi:10.1186/s13287-023-03481-7)
Supplement: Supplementary file 5 — Additional file 5: Fig. S5. Mouse GVHD clinical scoring system. Assessment of disease severity in mice based on fur texture, movements, posture, body weight loss and skin integrity. [file 13287_2023_3481_MOESM5_ESM.pdf]

Supplementary Figure 5

Parameter and score	0	1	2
**Fur texture (FT)**	Normal	Mild ruffling	Severe ruffling
**Activity/Movement (AM)**	Normal	Mild decrease	Severe decrease
**Posture (P)**	Normal	Hunching at rest	Severe hunching
**Body weight loss (BW)**	<10%	10-25%	>25%
**Skin integrity (SK)**	Normal	Scaling of paw/tail	Obvious denuded
