# Supplementary material for: Expansion of human bone marrow-derived mesenchymal stromal cells with enhanced immunomodulatory properties
Source: Stem Cell Res Ther. 2023 Sep 19;14:259. doi: 10.1186/s13287-023-03481-7 (PMC10510228; doi:10.1186/s13287-023-03481-7)
Supplement: Supplementary file 6 — Additional file 6: Fig. S6. Human CD45 (hCD45) blood cell count in GVHD mice at indicated day post-PBMC injection. hCD45 blood cell count in µl of blood for GVHD-bearing mice with saline (n=5), MSCLC (n=5) and MSCHC (n=4) on Day 17, 24, 28, 35, 42 post-PBMC injection. [file 13287_2023_3481_MOESM6_ESM.pdf]

Supplementary Figure 6

Day post PBMC injection	Saline (n = 5)	MSC_{LC} (n = 5)	MSC_{HC} (n = 4)
	Mean ± SEM of hCD45 count per µl blood		
D17	1545 ± 614.1	1801 ± 390.3	1276 ± 347.8
D24	5283 ± 3506	7676 ± 2659	1357 ± 544.9
D28	5618 ± 1790	9236 ± 3578	2086 ± 369.3
D35	5651 ± 2162	14367 ± 9578	3979 ± 1399
D42	3074 ± 776.9	9155 ± 4271	8777 ± 2828
